# Engraftment and morphological development of vascularized human iPS cell-derived 3D-cardiomyocyte tissue after xenotransplantation

**DOI:** 10.1038/s41598-017-14053-0

**Published:** 2017-10-20

**Authors:** Hirokazu Narita, Fumiaki Shima, Junya Yokoyama, Shigeru Miyagawa, Yoshinari Tsukamoto, Yasushi Takamura, Ayami Hiura, Ken Fukumoto, Tomohiro Chiba, Seiji Watanabe, Yoshiki Sawa, Mitsuru Akashi, Hiroshi Shimoda

**Affiliations:** 1 Department of Anatomical Science, Hirosaki University Graduate School of Medicine, 5 Zaifucho, Hirosaki, Aomori, 036-8562 Japan; 2 Department of Frontier Biosciences, Osaka University Graduate School of Frontier Biosciences, 1-3 Yamadaoka, Suita, Osaka, 565-0871 Japan; 3 Department of Cardiovascular Surgery, Osaka University Graduate School of Medicine, 2-2 Yamadaoka, Suita, Osaka, 565-0871 Japan; 4 Kyowa Hakko Bio Co., LTD, 1-9-2 Otemachi, Chiyoda-ku, Tokyo, 100-0004 Japan

## Abstract

One of the major challenges in cell-based cardiac regenerative medicine is the *in vitro* construction of three-dimensional (3D) tissues consisting of induced pluripotent stem cell-derived cardiomyocyte (iPSC-CM) and a blood vascular network supplying nutrients and oxygen throughout the tissue after implantation. We have successfully built a vascularized iPSC-CM 3D-tissue using our validated cell manipulation technique. In order to evaluate an availability of the 3D-tissue as a biomaterial, functional morphology of the tissues was examined by light and transmission electron microscopy through their implantation into the rat infarcted heart. Before implantation, the tissues showed distinctive myofibrils within iPSC-CMs and capillary-like endothelial tubes, but their profiles were still like immature. In contrast, engraftment of the tissues to the rat heart led the iPSC-CMs and endothelial tubes into organization of cell organelles and junctional apparatuses and prompt development of capillary network harboring host blood supply, respectively. A number of capillaries in the implanted tissues were derived from host vascular bed, whereas the others were likely to be composed by fusion of host and implanted endothelial cells. Thus, our vascularized iPSC-CM 3D-tissues may be a useful regenerative paradigm which will require additional expanded and long-term studies.

## Introduction

Ischemic heart disease is responsible for many deaths worldwide^1^. Although various advanced therapies have been developed for the cardiac disorder, it is so far impossible to revive the function of the necrotic myocardium because of reduced ability of mature cardiac muscle cells to proliferate and regenerate^2,3^. Therefore, cell-based regenerative medicine using stem cells such as embryonic stem cells or induced pluripotent stem cells (iPSCs) has attracted attention as a new therapeutic strategy to restore cardiac function in severe heart failure^4–6^. Besides, establishment of the method to differentiate human iPSC-CMs^7,8^ and readiness of ethical issue clearance^9^ also facilitate application of iPSC-CMs to cardiac regenerative medicine.

Meanwhile, direct engraftment of a large amount of iPSC-CMs into the target area of the heart remains a significant problem as to how to supply oxygen and nutrients into the graft for survival of the iPSC-CMs^10,11^. To address this problem, a cell sheet of the iPSC-CMs has been developed and reported to cause a beneficial effect on cardiac function through a paracrine effect after implantation to the infarcted porcine heart^12^. However, this iPSC-CM sheet could not provide sufficient thickness to supplement the mechanical function of severely disordered myocardium and its long-term survival^12^. Therefore, development of three-dimensional (3D) iPSC-CM tissues covering degenerated cardiac structure and function has been recently studied^13,14^ to enhance the *in vivo* survival ratio of the implanted tissues.

Since myocardia is well known to need abundant oxygen and nutrients to maintain their functions and structures, it is readily anticipated that thick iPSC-CM tissue implants result in their shortage within the implants, only depending on passive diffusion from the surrounding tissue. This is likely to eventuate in severe tissue damages involving cellular necrosis and a low cell survival ratio^15,16^. Thus, equipment of vasculature supplying nutrient and oxygen to the 3D iPSC-CM tissue is probably key challenge in its successful engraftment. Several studies have attempted to furnish the vasculature to the implanted iPSC-CMs through angiogenesis from the host tissue in the experiments using a stack of iPSC-CM sheets^14,17^ or matrigel-based tissue containing iPSC-CMs, endothelial cells and their mural cells^18^, but raised the issues on prompt and effective blood supply underlying conservation of the cellular activity and tissue development^14,17,18^, frequent surgical invasions^14^ and preparation of artificial scaffold^18^.

Our research group has recently developed a cell-accumulation technique that enables construction of 3D fibroblast tissue by coating extracellular matrix (ECM) nanofilms such as fibronectin onto single cell surfaces without an artificial scaffold^19^ and also succeeded in constructing a vascular network within the 3D fibroblast tissue by using this technique^19–21^. In addition, we have recently improved this cell-accumulation technique to fabricate a 3D iPSC-CM tissue with synchronous and periodic beating and found that the introduction of normal human cardiac fibroblasts (NHCFs) into iPSC-CM tissues plays an important role in modulating organization and synchronous beating depending on the proportion of NHCFs^22^.

In order to evaluate applicability of our vascularized iPSC-CM 3D-tissues as an implantation material for regenerative medicine, the present study demonstrates their morphological characteristics and alteration through implantation to the cardiac infarction model.

## Results

### Vascularized iPSC-CM 3D-tissue in *in vitro*

#### Light microscopy

**Figure 1:**
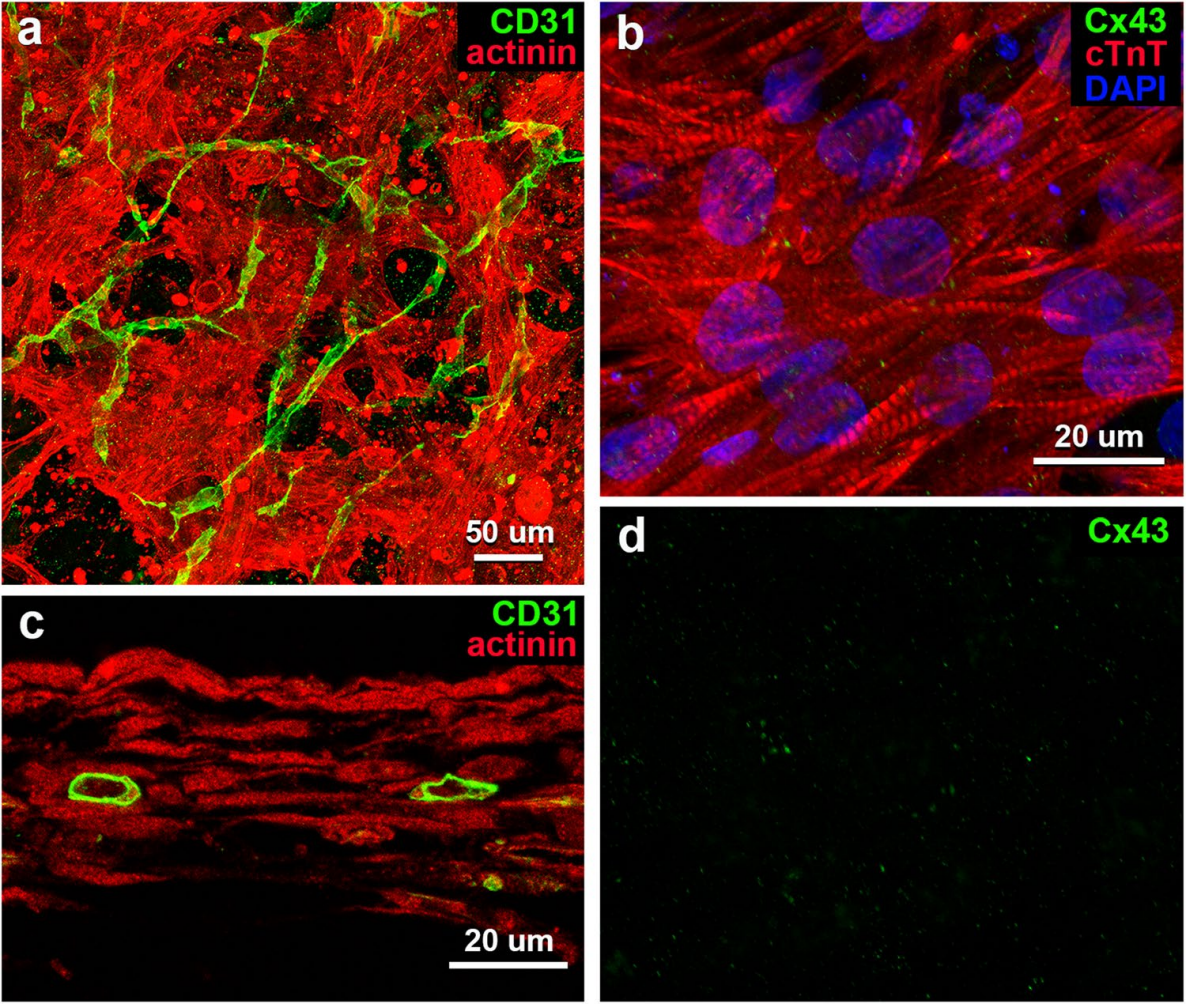
Light microscopic images of whole-mount preparations (**a**,**b**) and a tissue section (**c**) of vascularized iPSC-CM 3D-tissues 4 days after construction. (**a**,**c**) Immunostaining for sarcomeric α-actinin (red) and CD31 (green) shows a meshwork of iPSC-CMs preparing myofibrils and a network of endothelial tubes with lumens. (**b**) Immunostaining for cTnT (red) and Cx43 (green) shows sarcomeres in iPSC-CMs, and a small amount of reaction products for Cx43 on the cells. Cellular nuclei are stained with DAPI. (**d**) An image of reaction products for Cx43 distilled from **b**.

Immunohistochemical analysis of our fabricated iPSC-CM 3D-tissues (ratio of cell numbers: iPSC-CM:NHCF:HCMVEC = 7.5:2.5:1) demonstrated a three-dimensional meshwork of iPSC-CMs being immunopositive for cardiomyocyte biomarkers such as sarcomeric α-actinin and cardiac troponin T (cTnT) (Fig. 1a,b). The cardiomyocytes elongated and branched in every direction to connect to each other (Fig. 1a,b), but immunolocalization of connexin 43 (Cx43), a representative protein constituting a gap junction, was scanty at their connective sites (Fig. 1b,d). Although the cells also developed some myofirils with distinct sacomeres in their sarcoplasm, alignment of the myofibrils was irregular in this *in vitro* condition (Fig. 1b). Meanwhile, the cardiac endothelial cells loaded into the 3D-tissues built many CD31-immunopositive tubes with luminal structures extending a network around the iPSC-CMs meshwork (Fig. 1a,c).

#### Transmission electron microscopy

**Figure 2:**
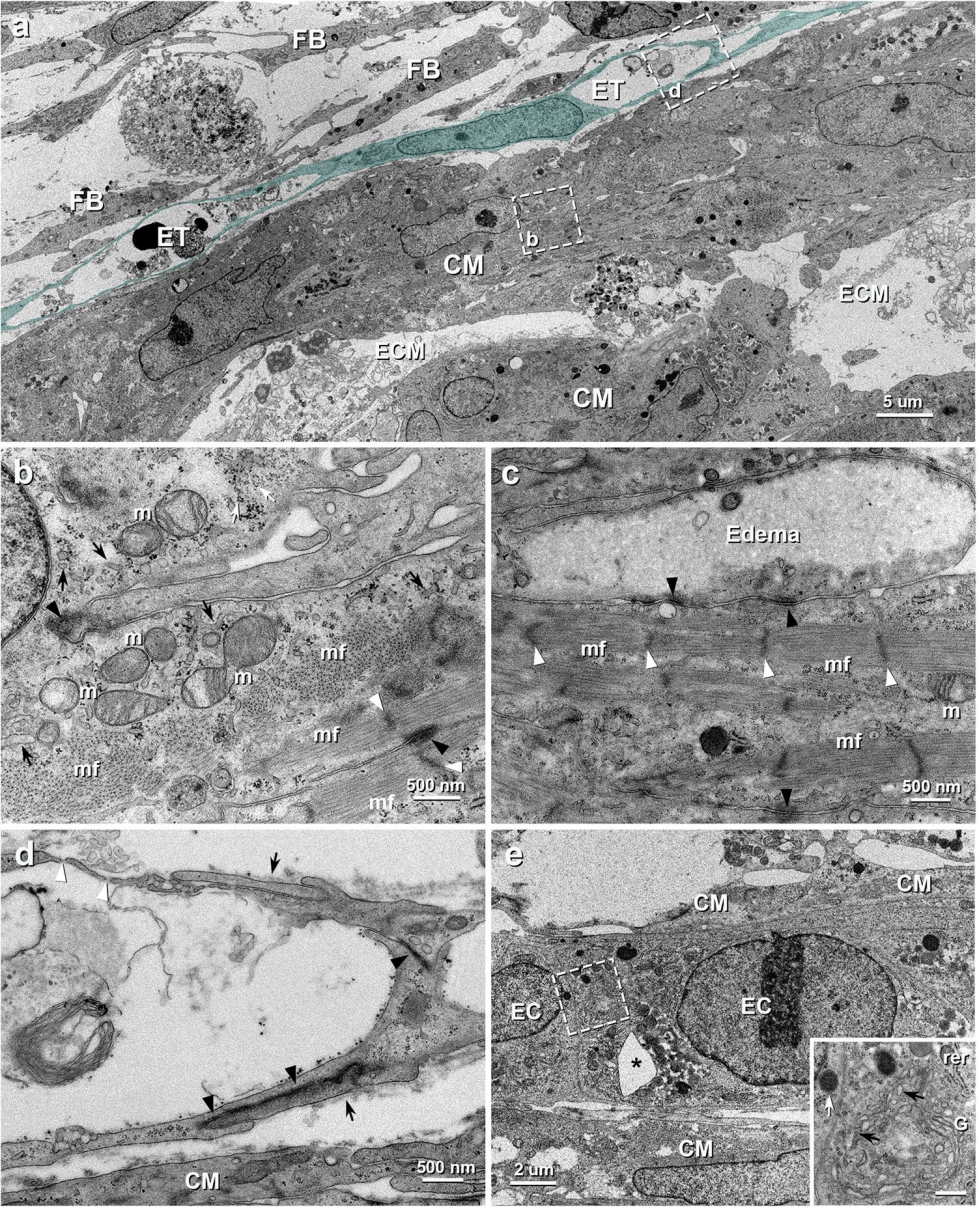
TEM images of vascularized iPSC-CM 3D-tissues 4 days after construction. (**a**) The iPSC-CMs (CM) are assembled to contact with each other within some amount of extracellular matrices (ECM) and the endothelial (blue) tubes (ET) extends along the iPSC-CMs. FB, fibroblast. (**b**) Higher magnification of the boxed area in **a**. Numerous myofilaments assemble to form myofibrils (mf), whereas some myofilaments with poor connectivity (white arrow) are also seen. Small mitochondria (m) with a few cristae are sparsely distributed within sarcoplasm. Black arrows, white and black arrowheads indicate sarcoplasmic reticula, Z-bands and desmosomes between the adjacent iPSC-CMs, respectively. (**c**) Higher magnification of the contact area of the iPSC-CMs. Many desmosomes (black arrowheads) are seen between the cells, but gap junction is indistinguishable. The cell at the top shows intracellular edema. White arrowheads indicate Z-bands of myofibrils (mf). m, mitochondria. (**d**) Higher magnification of the boxed area in **a**. The endothelial cells connect to each other by overlapping (arrows) with junctional complex (black arrowheads) to form vascular lumen. The endothelial pores (white arrowheads) are also seen. (**e**) A insertion of extracellular tissue (*) is seen in the endothelial cords (EC). CM, iPSC-CM. *Inset:* Higher magnification of the boxed area in **e**. The endothelial cell contains abundant rough endoplasmic reticula (rer), coated vesicles (black arrows), Golgi apparatuses (G) and lysosomes (white arrow). Scale bar: 500 nm.

The fine structure of the iPSC-CM tissues was clearly demonstrated by transmission electron microscopy (TEM). The irregularly-shaped iPSC-CMs aggregated to a few layer and contacted with each other by adhering apparatus consisting predominantly of desmosomes (Fig. 2a–c). However, distinctive structures evidencing gap junctions were not found in the present TEM observation covering 210,000 μm^2^ each of the three 3D-tissues, though a few amounts of the reaction products for Cx43 were found under light microscopy. Each manufactured cardiomyocytes contained some myofibrils, small and oval mitochondria with a few cristae, sarcoplasmic reticula, glycogen granules and lysosomes around their elliptical nucleus (Fig. 2a,b). The myofibrils were randomly arrayed within the cytoplasm, and many free myofilaments, which were not developed into myofibrils, were also observed (Fig. 2b). In addition, intracellular edema-like lesions of various degrees were recognized in some iPSC-CMs (Fig. 2c).

TEM examination of the 3D-tissue further disclosed many tubular structures consisting of the ECs around the iPSC-CMs and their developing process (Fig. 2d,e). The flattened and elongated ECs developed such cell organelles containing coated vesicles, rough endoplasmic reticula, Golgi apparatuses, and lysosomes (Fig. 2e) as seen in vascular endothelial cells in living body and interconnected by overlapping with adherens junctions and desmosomes to form capillary-like tubes (Fig. 2a,d). The endothelial pores being like fenestrations were also shown in some parts of the vascular tubes (Fig. 2d). The extracellular matrices prepared the vessels a basal lamina-like structure in parts (Fig. 2d). Some aggregated ECs formed funicular structures with insertion of extracellular tissue pillar (Fig. 2e), indicating one of the angiogenic events^23^.

### Vascularized iPSC-CM 3D-tissues after implantation in rat infarcted hearts

#### Light microscopy

**Figure 3:**
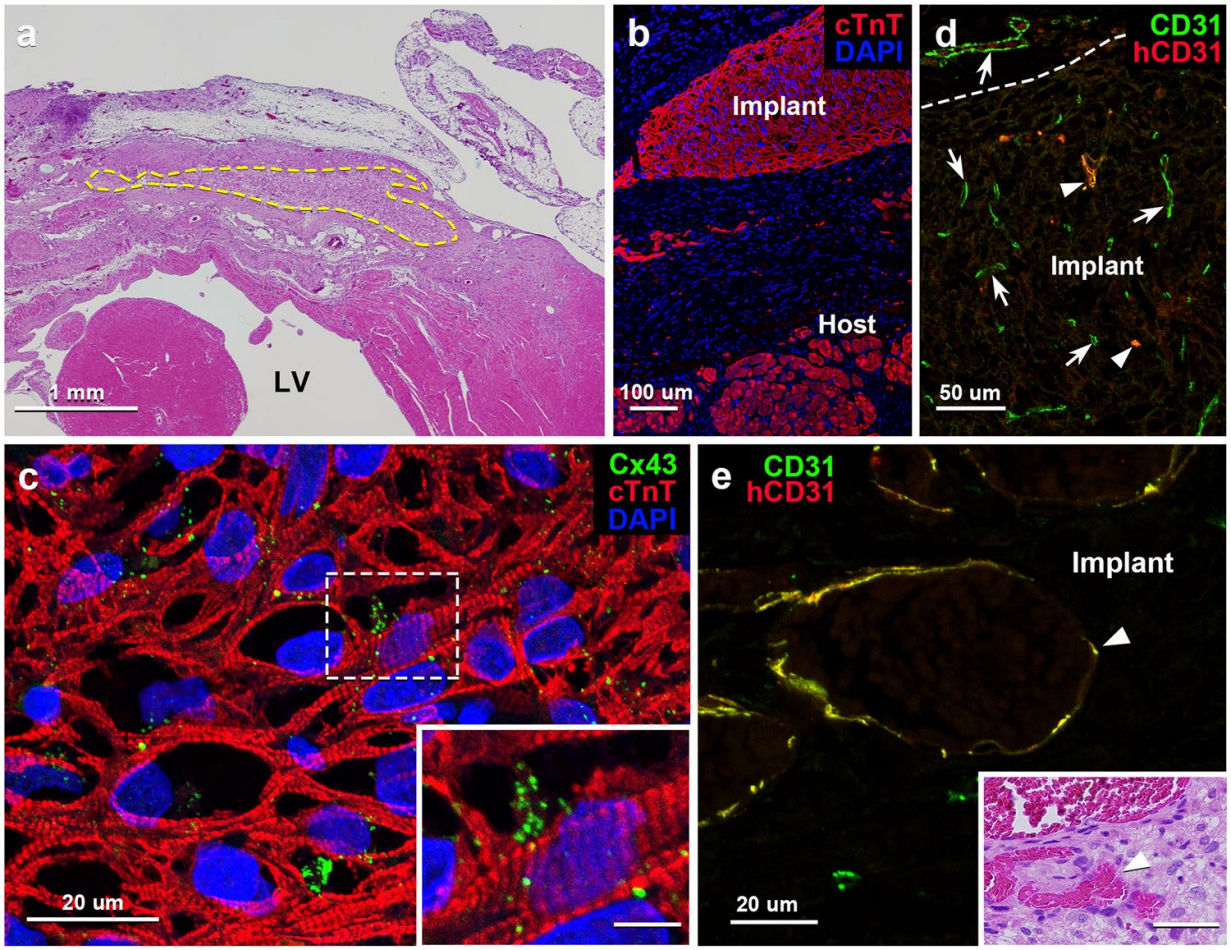
Light microscopic images of tissue sections of vascularized iPSC-CM 3D-tissues 28 days after implantation to rat infarcted heart. (**a**) Transverse section of rat heart implanted with the 3D-tissue with hematoxylin-eosin (H.E.) staining. The area encircled by the dotted line indicates the implanted tissue in the infarcted left ventricle (LV). (**b**) Immunostaining for cTnT (red) depicts cardiomyocytes both in the implant and host residual myocardium. Cellular nuclei are stained with DAPI. (**c**) Immunostaining for cTnT (red) and Cx43 (green) shows a fine meshwork of iPSC-CMs with substantially-ranged sarcomeres and many reaction products for Cx43 on them. Cellular nuclei are stained with DAPI. *Inset:* Higher magnification of the boxed area in c. Scale bar: 5 µm. (**d**) Immunostaining for human CD31 (red) and multi-species CD31 (green) shows many host-derived vessels (arrows) immunopositive only for multi-species CD31 and a few implant-derived vessels (arrowheads) immunopositive both for human and multi-species CD31 (yellow) within the implant. The dotted line indicates the border between the host and implant tissue. (**e**) Higher magnification of an implant-derived vessel (arrowhead) immunopositive both for human and multi-species CD31 (yellow). *Inset:* The H.E.-stained tissue section adjacent to e shows blood contents within the implant-derived vessels (arrowhead). Scale bar: 40 µm.

The iPSC-CM 3D-tissues implanted on the left ventricle of the experimentally-infarcted rat heart were well survived until 28 days after the operation (Fig. 3a,b and Supplementary Fig. 1), and the implanted tissues significantly increased in thickness (261 ± 64 µm on day 28 following operation) as compared to those in *in vitro* (69 ± 20 µm; p < 0.05) (Supplementary Fig. 2a,b). The iPSC-CMs in the implanted tissues revealed abundant myofibrils with cTnT-immunoreactivity, of which aspects were similar to those in the *in vitro* tissues, until the endpoint of the experiment (Fig. 3c). Immunoreaction products for gap junction protein, Cx43, on the contrary, accumulated on the iPSC-CMs in this implanted condition (Fig. 3c). No neoplasms were recognized at least throughout the experimental period.

**Figure 4:**
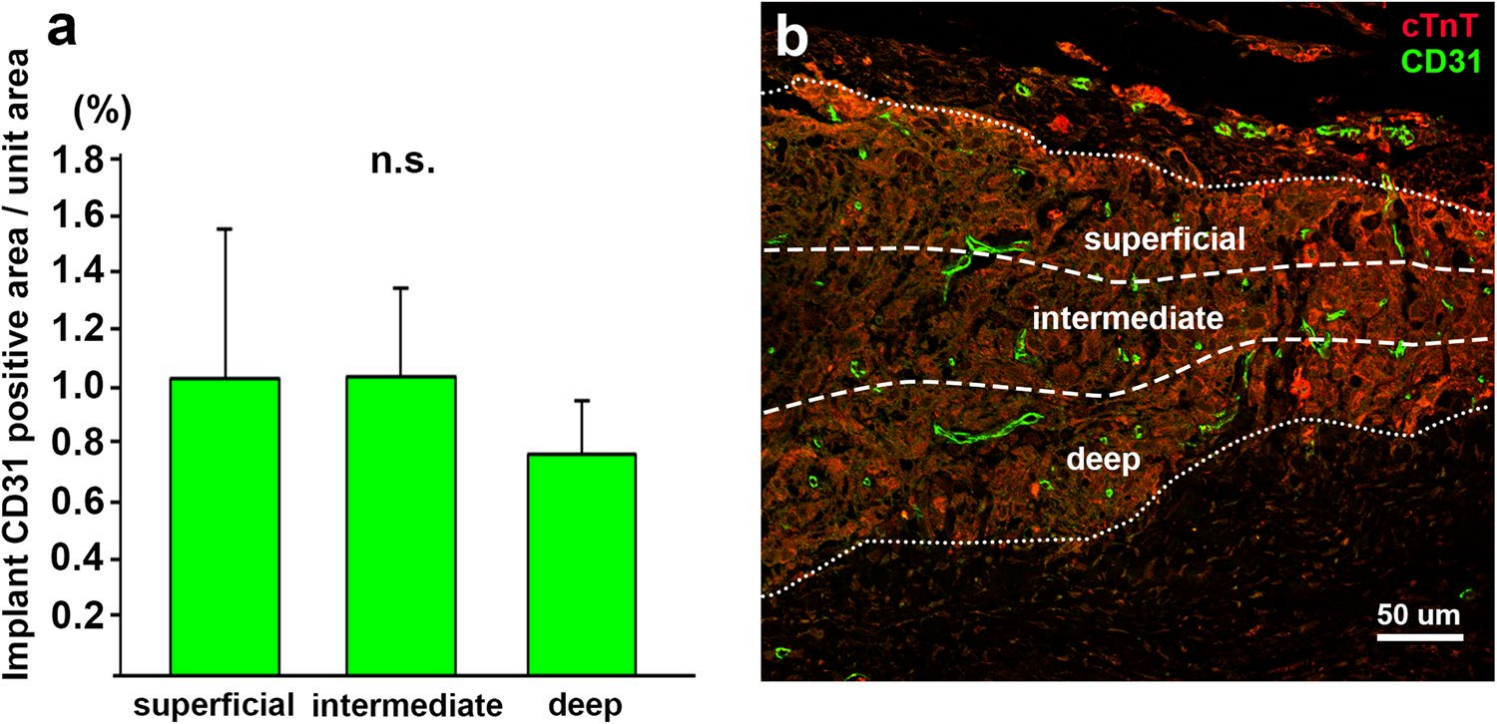
Comparison of vascular distribution in iPSC-CM 3D-tissues 28 days after implantation to rat infarcted heart. (**a**) The ratio of the CD31-immunopositive vascular area to unit area in the deep region (0.76 ± 0.19%; n = 3, total 6 fields) is lower than that in the intermediate (1.03 ± 0.31%; n = 3, total 6 fields) or superficial (1.04 ± 0.52%; n = 3, total 6 fields) region within the implant, though there is no statistical difference among each region. (**b**) A representative image of three divided regions in the implant and vascular distribution in each region. Immunostaining for cTnT (red) and multi-species CD31 (green) on a transverse section of the iPSC-CM 3D-tissue implanted to the infarcted rat heart. The pericardium is located at the top of the image.

Our 3D-tissues displayed a wide distribution of blood vessels immunopositive for multi-species CD31 28 days following implantation, but the vascular density in the deep portion away from rat epicardium was lower than that in the superficial portion (Fig. 4). Double immunostaining with anti-human and anti-multispecies CD31 antibodies further enabled to discriminate between implant-derived human and host-derived rat blood vessels in the implanted tissues: most of the vessels were derived from host vessels immunoreactive only for multi-species CD31, and a small number of the vessels, which were immunopositive both for human and multi-species CD31, were of implanted human origin (Fig. 3d,e). Numerous blood vessels containing blood cells appeared within the implants and the blood vascular area in the 3D-tissues was significantly increased after implantation (p < 0.05), with the fact that the area were still less than that in rat cardiac ventricle (Supplementary Fig. 3).

#### Transmission electron microscopy

**Figure 5:**
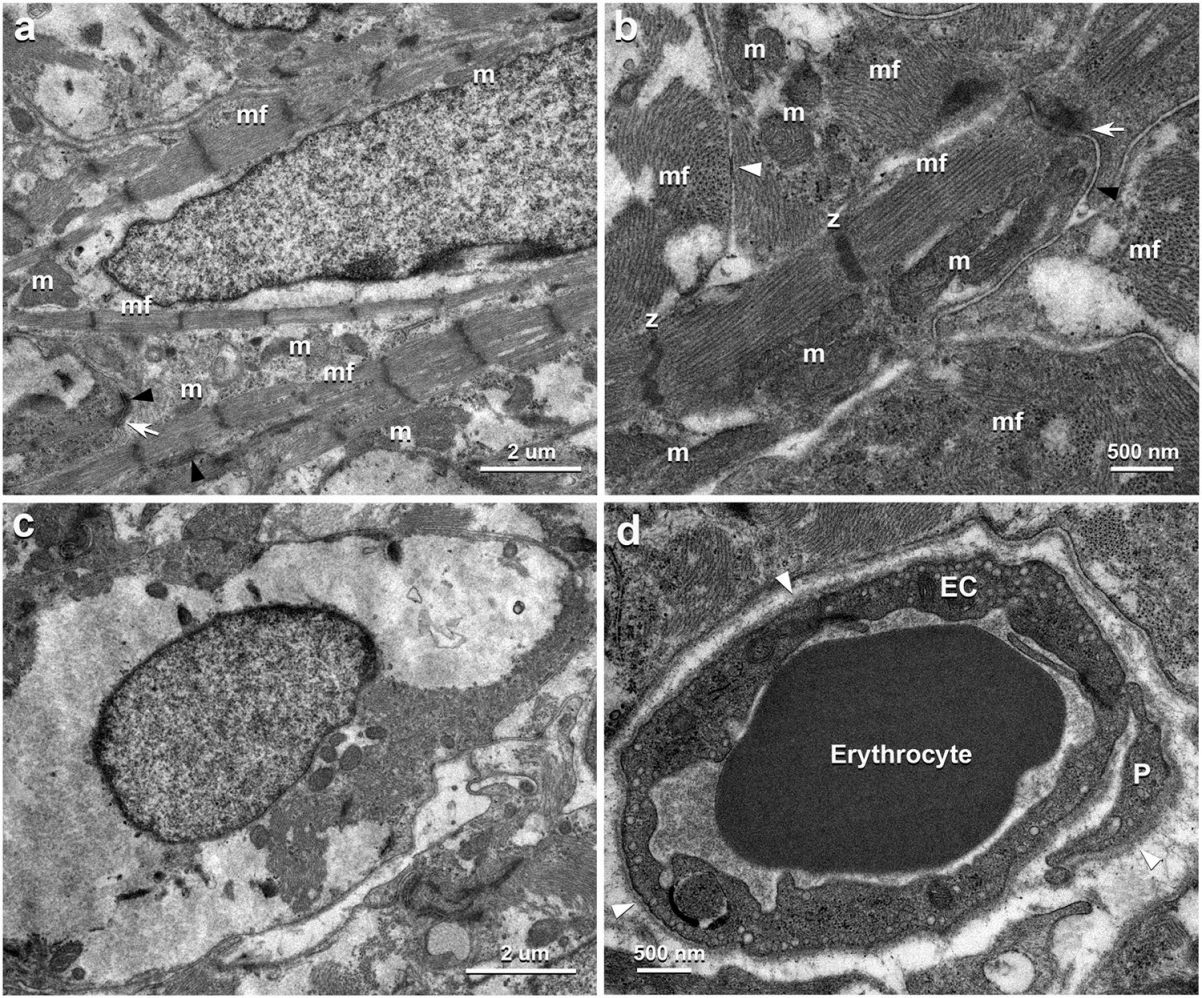
TEM images of vascularized iPSC-CM 3D-tissues 28 days after implantation to rat infarcted heart. (**a**) The iPSC-CM forms numerous myofilaments into substantial myofibrils (mf), which distribute densely within the sarcoplasm. Many oval mitochondria (m) are seen ranging along the myofibrils. The arrow and arrowheads indicate adherens junction and desmosomes between adjacent iPSC-CMs, respectively. (**b**) The contact area between the adjacent iPSC-CMs shows substantial intercalated disk equipping gap junction (white arrowhead), adherens junction (white arrow) and desmosome (black arrowhead). The mitochondria (m) show many lamellar cristae. mf, myofibril; z, Z-band. (**c**) The iPSC-CMs in the deep region shows intracellular edema. (**d**) The endothelial cells (EC) form continuous capillary equipping substantial continuous basal lamina (white arrowheads) and pericyte (P). An erythrocyte are also seen in the vascular lumen.

Under TEM in the implanted 3D-tissues, the iPSC-CMs basically exhibited similar cytological features to those in *in vitro* 3D-tissue, whereas thickened myofibrils tended to be in parallel and numerous cylindrical and/or elliptical mitochondria with many lamellar cristae were closely arranged along the myofibrils after implantation (Fig. 5a,b and Supplementary Fig. 4). The gap junctions were also disclosed at many contact sites between the iPSC-CMs, as well as numerous desmosomes and fasciae adherens, after implantation at an electron microscopic level (Fig. 5b), as shown by immunohistochemistry for Cx43 (Fig. 3c). However, some iPSC-CMs revealed such degenerative aspects as intracellular edema in the deep portion of the implants (Fig. 5c). TEM examination further demonstrated a lot of continuous-type capillaries equipped with substantial basal laminae and pericytes (Fig. 5d).

## Discussion

This is the first study reporting the morphological features of vascularized iPSC-CM 3D-tissues fabricated by our peculiar cell-accumulation technique in *in vitro* condition and following engraftment. The iPSC-CMs and endothelial cells organized into networked constructions with spatial extension within *in vitro* 3D-tissues. Most iPSC-CMs, however, impressed an immature morphological profile in this context, because their cell organelles were small and sparse as seen in embryonic myocardium^24,25^ (Fig. 2a,b and Supplementary Fig. 5). The similar findings have been reported in the previous studies on the iPSC-CMs cell sheets^14,26^. The endothelial network, meanwhile, were composed of tubular structures with distinct lumens but insufficiently encompassed by basal lamina. Since formation of basal lamina is thought to indicate the degree of maturation and stabilization in native capillaries^27^, the endothelial tubes are probably regarded as angiogenic immature vessels. The endothelial cord-like structures with insertion of tissue pillar indicating an angiogenic process^23^ also support this idea.

Despite displaying a coordinated contraction^22^, our iPSC-CM tissues demonstrated only a few immunoreaction products for Cx43 and no structures indicating the existence of gap junctions, being regarded as a specific apparatus providing synchronous cardiac contraction^28^, were found under TEM until 4 days after construction. Several previous studies investigating iPSC-CM adhesions have described a sporadic expression of Cx43 on the plasmalemma^16,17,29^, whereas Gherghiceanu *et al*.^30^ have reported no obvious gap junction on 31-days cultured iPSC-CMs under TEM, in spite of their coordinated contraction, which may also be related to the recognized technical challenges of immunostaining gap junctions in cardiac tissues. These inconsistencies might result from the possibility that the Cx43 protein begins to be expressed before completion of gap junctions, along with the immaturity of the iPSC-CMs. As for gap junctions, their accumulation has been reported not to be noticeable before birth^31^, and Gutstein, D. E. *et al*.^32,33^ have suggested a dispensability of gap junctions because of normal cardiac function developed in cardiac-specific Cx43 conditional knock out mice at two weeks of age. Therefore, ultrastructural absence of gap junctions in our iPSC-CM tissue in *in vitro* might be associated with its immaturity.

Following implantation, our 3D-tisseus were well survived in long term and thickened as compared with those in *in vitro* (Supplementary Fig. 2a,b). Some iPSC-CMs expressed cell proliferation marker, Ki67, even at 28 days after implantation (Supplementary Fig. 2c), so that their proliferative activity presumably contribute to an increase of the implanted tissue volume, though it cannot be denied that the tissue has been deformed. The cells also showed a tendency of thickened myofibrils to range in parallel and of enlarged mitochondria to distribute along the myofibrils (Fig. 5a,b and Supplementary Fig. 4), as seen in mature living myocardium. Their ultrastructural alteration implies that the iPSC-CMs in our implanted 3D-tissues hold a potential to mature rapidly within 28 days as compared to those in stacks of iPSC-CM cell sheets at 6 months after subcutaneous implantation^14^.

Although the implanted iPSC-CMs are likely to have the developmental capability as described above, the deeper portion of the implants away from the host epicardium showed several degenerative iPSC-CMs in correspondence to the lower vascular density. This suggests that vascularization in the implanted tissues is crucial in establishment of the iPSC-CMs in the host heart, though passive diffusive flow from the surrounding host tissue possibly effects the implantation. Our additional experimental data demonstrated that 3D-tissues of multilayered fibroblasts without endothelial cells contained no vessels 14 days after subcutaneous implantation, whereas those with endothelial tubes exhibited a marked extension of host blood vessels into the implants and connections between the host and implanted vessels to form circulation promptly (Supplementary Fig. 6). Some previous findings have also reported the application of endothelial tubes embraced in implants to provide blood supply^34,35^. The blood vessels in our implanted tissues further displayed matured vessels as continuous capillaries in their ultrastructure. Thus, investment of vascular network in the iPSC-CM 3D-tissues is likely to play a pivotal role in successful engraftment of the iPSC-CMs. Nevertheless, the vascular density in the implants was approximately one fourth of that in native rat cardiac ventricles even 28 days after implantation (Supplementary Fig. 3), and the implanted region away from pericardium often showed degenerative iPSC-CMs. This supposedly indicates necessity of denser vasculature in the implants to yield sufficient blood supply.

The present study demonstrated the precise morphological characteristics of our peculiar iPSC-CM 3D-tissues with vascular network and their alteration through implantation on the myocardial infarction model. Some previous reports have described mechanical and physiological effects of iPSC-CM implantation^18,36^, but no reports, to our own knowledge, have investigated as regards three-dimensionally fabricated iPSC-CM tissue equipped with vasculature. Our present findings might provide a capability of our vascularized iPSC-CM 3D-tissue as a biomaterial proper for regenerative medicine against degenerative cardiac diseases involving cardiac infarction, though further and long-term examinations for mechanical and functional recovery including incidence of arrhythmia and such improvements as development of more elaborate vasculature and vigorous iPSC-CMs are required for clinical trials^36,37^.

## Methods

### Preparation of iPSC-CMs

#### Materials

Mitomycin C-inactivated mouse embryo fibroblast feeder cells, human recombinant FGF-2 and 1-thioglycerol, dispase and TeSR-E8 were purchased from Millipore Co. (MA, USA), Sigma-Aldrich Inc (MO, USA), Roche Diagnostics (Basel, Switzerland) and STEMCELL Technologies (Vancouver, Canada), respectively. Knockout-DMEM/F12, knockout serum replacement, 2-mercaptoethanol, MEM non-essential amino acids, L-glutamine and StemPro-34 were purchased from Thermo Fisher Scientific Inc. (MA, USA). L-ascorbic acid 2-phosphate trisodium salt, IWR-1-endo and Y-27632 were purchased from Wako Pure Chemical Industries (Osaka, Japan). Recombinant human proteins of BMP-4, activin A and VEGF were purchased from R&D systems Inc (MN, USA).

#### Procedure for preparing iPSC-CMs

The human iPS cell line, 253G1^38^ was routinely maintained on mitomycin C-inactivated mouse embryo fibroblast feeder cells in knockout serum replacement (KSR)-based medium supplemented with 4 ng/ml FGF-2. KSR-based medium consisted of knockout-DMEM/F12 medium, supplemented with 20% (v/v) KSR, 0.1 mM 2-mercaptoethanol, MEM non-essential amino acids, and 2 mM L-glutamine. Cells were passaged as small clumps every 6–7 days using 1 mg/ml dispase. The clumps and single cells after accutase treatment were suspended in 30 ml TeSR-E8 containing 10 μM Y-27632 and seeded into a 30 ml single-use bioreactor (ABLE Corporation & Biott Co., Japan). The agitation rate was 55 rpm. After one day, the medium was changed to TeSR-E8. After day 3, EBs formed in bioreactor were cultured in StemPro-34 medium containing 50 µg/ml L-ascorbic acid 2-phosphate trisodium salt, 2 mM L-glutamine and 400 μM 1-thioglycerol. The following growth factors and small molecules were used at the corresponding days: days 3–4, 0.5 ng/ml BMP-4; days 4–9, 10 ng/ml BMP-4, 5 ng/ml bFGF, 3 ng/ml activin A; days 9–11, 4 uM IWR-1; after day 11, 5 ng/ml VEGF and 10 ng/ml FGF-2. At days, 6, 8, 11 and 13, the culture medium was exchanged. iPSC-CMs at day 15 were employed in this paper.

### Construction of three-dimensional iPSC-CM tissue containing vascular network

#### Materials

All of the reagents were used without further purification. Fibronectin (FN) from human plasma, gelatin (G), Dulbecco’s modified eagle medium (DMEM), fetal bovine serum and the cell culture insert with a 0.4 or 3 µm pore membrane was purchased from Sigma-Aldrich (MO, USA), Wako Pure Chemical Industries (Osaka, Japan), Nacalai Tesque (Kyoto, Japan), Life Technologies (CA, USA) and Corning (NY, USA), respectively. Normal human cardiac fibroblasts (NHCFs), normal human cardiac microvascular endothelial cells (HCMVECs), cardiac fibroblast growth medium (FGM-3) and endothelial growth medium (EGM-2 MV) were further purchased from Lonza (NJ, USA).

#### Procedure for constructing three-dimensional iPSC-CM tissue containing vascular network

The iPSC-CM 3D-tissue containing vascular network were constructed employing filtration Layer-by-Layer (LbL) technique that enabled coating of individual cells with ECM-nanofilms, with minimal damage^22^. Briefly, iPSC-CMs and NHCFs were suspended in 0.2 mg/ml FN/PBS or G/PBS solution using a 6 well culture insert (3 µm pore size), and incubated for 1 min at room temperature using a plate mixer (MixMate, Eppendorf, Germany). Between each coating step, the cells were washed with PBS. These coating procedures were performed total 9 times (FN: 5 times, G: 4 times), and the cells whose surface was coated with FN-G nanofilm were collected by suspending in medium.

To construct iPSC-CM 3D-tissue with vascular network, the FN-G-coated iPSC-CMs and NHCFs, and non-coated HCMVECs were mixed and seeded onto a 24 or 12 well culture insert. In this study, we fabricated the tissue with the ratio of iPSC-CMs and NHCFs to HCMVECs being 7.5:2.5:1 because it showed better beating of iPSC-CMs than those fabricated with another ratio^22^. Thus, we totally seeded 1.1 × 10^6^ or 3.7 × 10^6^ cells onto the 24 or 12 well culture insert respectively, which correspond to about 10 layers. These inserts were put into 6 well plate, added 2 ml of culture medium, and incubated for 1 h at 37 °C in 5% CO_2_. An additional 10 ml of medium was added to each well, and the samples were then incubated for 4 days. After cultured for 4 days, the samples which fabricated in the 24 and 12 well culture insert were applied for analyses of their morphology in *in vitro* and after implantation, respectively. For comparison, we also cultured them for longer time, more than 1 week. However, the iPSC-CM tissues were shrunk to detach from the insert membrane.

### Infarcted heart model preparation and implantation of iPSC-CM tissue

All animal experiments were approved by Animal Research Committee of Osaka University. Animal care was conducted in compliance with the Guide for the Care and Use of Laboratory Animals prepared by the Institute of Animal Resources.

Female F344/NJcl-rnu/rnu rats at 7 weeks of age (Crea, Tokyo, Japan) were generally anesthetized with inhalation of isoflurane (1.5%; Mylan Inc., Tokyo, Japan) and intubated and mechanically ventilated. The proximal left anterior descending artery at 2 mm below the left appendage was permanently ligated with a 6/0 polypropylene snare (Ethicon, Johnson & Johnson, USA) under left thoracotomy. Two weeks after the ligation, transthoracic echocardiography (ViVid i; GE Healthcare, WI, USA) with a 11.5-MHz transducer was performed, and heart failure model rats (LVEF < 50%) were selected (n = 3). Rats were anesthetized and the heart was re-exposed through left thoracotomy approach. The vascularized iPSC-CM 3D-tissue was placed on the surface of the left ventricle and was sutured with 7/0 polypropylene. At 28 days after the treatment, under anesthesia by 5% isoflurane inhalation, rats were sacrificed and the heart was promptly dissected.

### Histological analysis

iPSC-CM 3D-tissues in *in vitro* and resected rat hearts were fixed with 4% paraformaldehyde (PFA) in PBS. Some iPSC-CM tissues in *in vitro* were used for whole-mount fluorescent immunostaining, and the other iPSC-CM tissues and rat hearts were embedded with paraffin.

For whole-mount immunostaining, after incubated in PBS containing 0.3% Triton X-100 for one week and in 10% normal donkey serum (R&D systems) overnight, tissues were immersed in primary antibodies for one week at 4 °C. Following washing step, secondary antibodies was applied for 2 days at 4 °C.

For paraffin-embedded samples, sections (5 µm thickness) were prepared and stained with hematoxylin and eosin (H.E.). Fluorescent immunostaining was conducted in a similar method to previous reports^39,40^. Briefly, the deparaffinized sections were incubated in a 0.01 M citrate buffer (pH 6.0) at 121 °C for 15 min to retrieve antigenicity of the objective proteins. Subsequently, sections were incubated in 10% normal donkey serum for 15 minutes, in primary antibodies overnight, and then in secondary antibodies for 2 hours at room temperature.

For immunohistochemistry, mouse monoclonal anti-sarcomeric alpha actinin antibody (Abcam, Cambridge, UK), mouse monoclonal anti-cardiac troponin T antibody (Thermo Fisher Scientific, Waltham, USA), rabbit polyclonal anti-connexin43 antibody (Thermo Fisher Scientific), rabbit polyclonal anti-CD31 antibody (Abcam) and mouse monoclonal anti-human CD31 antibody (Dako, Cytomation, UK) were used for primary antibodies. Indocarbocyanine (Cy3)-conjugated donkey anti-mouse IgG (Jackson ImmunoResearch, West Groove, PA, USA) and fluorescein isothiocyanate (FITC)-conjugated donkey anti-rabbit IgG (Jackson ImmunoResearch) were applied as secondary antibodies. For fluorescent labeling of nucleus, 4, 6-diamidino-2-phenylindole (DAPI; Dojindo, Kumamoto, Japan) was used.

### Ultrastructural analysis

Previous reports were consulted for ultrastructural analyses using transmission electron microscopy (TEM)^41^. Briefly, iPSC-CM 3D-tissues in *in vitro* and resected rat hearts were fixed with 2.5% glutaraldehyde and 1% PFA in 0.1 M phosphate buffer (PB) containing 0.01% CaCl_2_ and MgCl_2_, and cut into small tissue pieces. Following post-fixation in 1% osmium teroxide solution for 1 h at 4 °C, these tissues were dehydrated in a graded ethanol series and embedded in Epon 812 (Nisshin EM, Japan). Ultra-thin sections were prepared at 70 nm thickness with an ultramicrotome (Reichert Ultracut S; Leica, Germany), and then electron staining with uranyl acetate and lead citrate was applied.

### Imaging and image processing

The images of H.E. staining and fluorescent immunostaining were obtained with a bright-field microscope (BX50; Olympus, Tokyo, Japan) and confocal laser scanning microscope (LSM 710; Carl Zeiss, Jena, Germany), respectively. TEM images were captured with a JEM1250 (JEOL, Tokyo, Japan). Images were processed using Photoshop CC (Adobe Systems, San Jose, USA).

### Statistical analysis

The thickness of the iPSC-CM tissues in *in vitro* (n = 3) and after implantation (n = 3) were measured from the acquired cross-sectional images. The implanted tissues in the sectional images (n = 3, total 6 fields) were evenly divided into three subdivisions, viz., superficial, intermediate and deep portion, and the CD31-immunopositive vascular area in each portion were measured using National Institutes of Health’s ImageJ software and then expressed as a percentage. These data were presented as means ± standard deviations. Comparisons between each tissue thickness and of ratio of the CD31-positive-cell-area per unit area among in each subdivision of the implants were performed by Student’s unpaired t-test and Tukey’s test, respectively, at a significance level of 0.05 using SPSS 12.0 J for Windows software package (SPSS Japan Inc., Tokyo, Japan).

## Electronic supplementary material

Supplementary information
