# Supplementary material for: Engraftment and morphological development of vascularized human iPS cell-derived 3D-cardiomyocyte tissue after xenotransplantation
Source: Sci Rep. 2017 Oct 20;7:13708. doi: 10.1038/s41598-017-14053-0 (PMC5651879; doi:10.1038/s41598-017-14053-0)
Supplement: Supplementary file 1 — Supplementary information [file 41598_2017_14053_MOESM1_ESM.doc]

**Engraftment and morphological development of vascularized human iPS cell-derived 3D-cardiomyocyte tissue after xenotransplantation**

Authors:

Hirokazu Narita^1^, Fumiaki Shima^2^, Junya Yokoyama^3^, Shigeru Miyagawa^3^, Yoshinari Tsukamoto^2^, Yasushi Takamura^2^, Ayami Hiura^2^, Ken Fukumoto^4^, Tomohiro Chiba^1^, Seiji Watanabe^1^, Yoshiki Sawa^3^, Mitsuru Akashi^2^, Hiroshi Shimoda^1*^

Affiliations and contact information:

^1^ Department of Anatomical Science, Hirosaki University Graduate School of Medicine, 5 Zaifucho, Hirosaki, Aomori 036-8562, Japan

^2^ Department of Frontier Biosciences, Osaka University Graduate School of Frontier Biosciences, 1-3 Yamadaoka, Suita, Osaka 565-0871, Japan

^3^ Department of Cardiovascular Surgery, Osaka University Graduate School of Medicine, 2-2 Yamadaoka, Suita, Osaka 565-0871, Japan

^4^ Kyowa Hakko Bio Co., LTD, 1-9-2 Otemachi, Chiyoda-ku, Tokyo 100-0004, Japan

^*^ Corresponding author:

Hiroshi Shimoda

Department of Anatomical Science, Hirosaki University Graduate School of Medicine, 5 Zaifucho, Hirosaki, Aomori 036-8562, Japan

Tel: +81-172-39-5004

E-mail: *hshimoda@hirosaki-u.ac.jp*

**
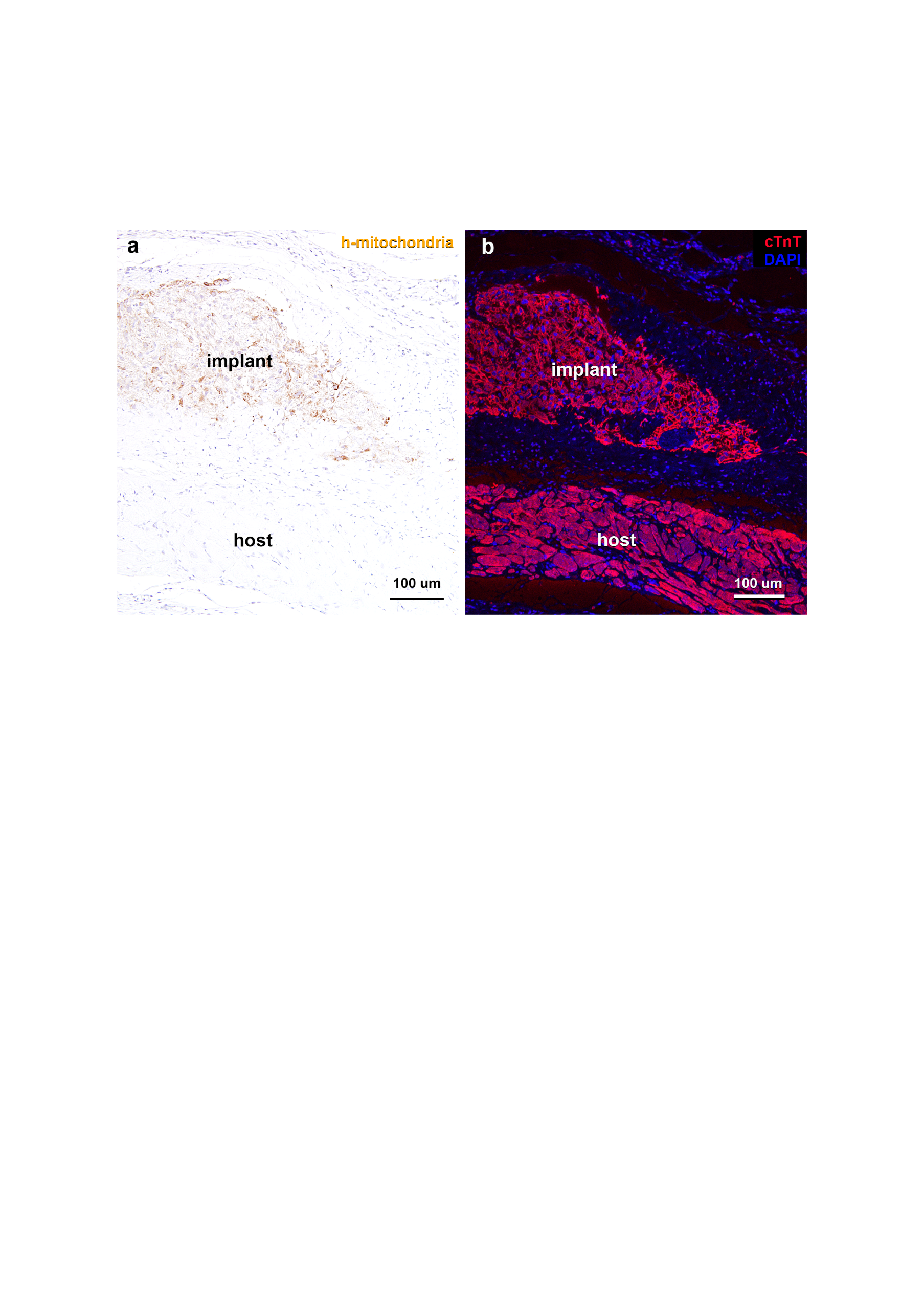
**

**Supplementary Figure 1 │Immunostaining for human mitochondria (brown in a) and cTnT (red in b) on adjacent tissue sections of vascularized iPSC-CM 3D-tissues 28 days after implantation to rat infarcted heart.** The human mitochondria-immunoreactivity are seen in the implanted tissue but not in host myocardium.

**
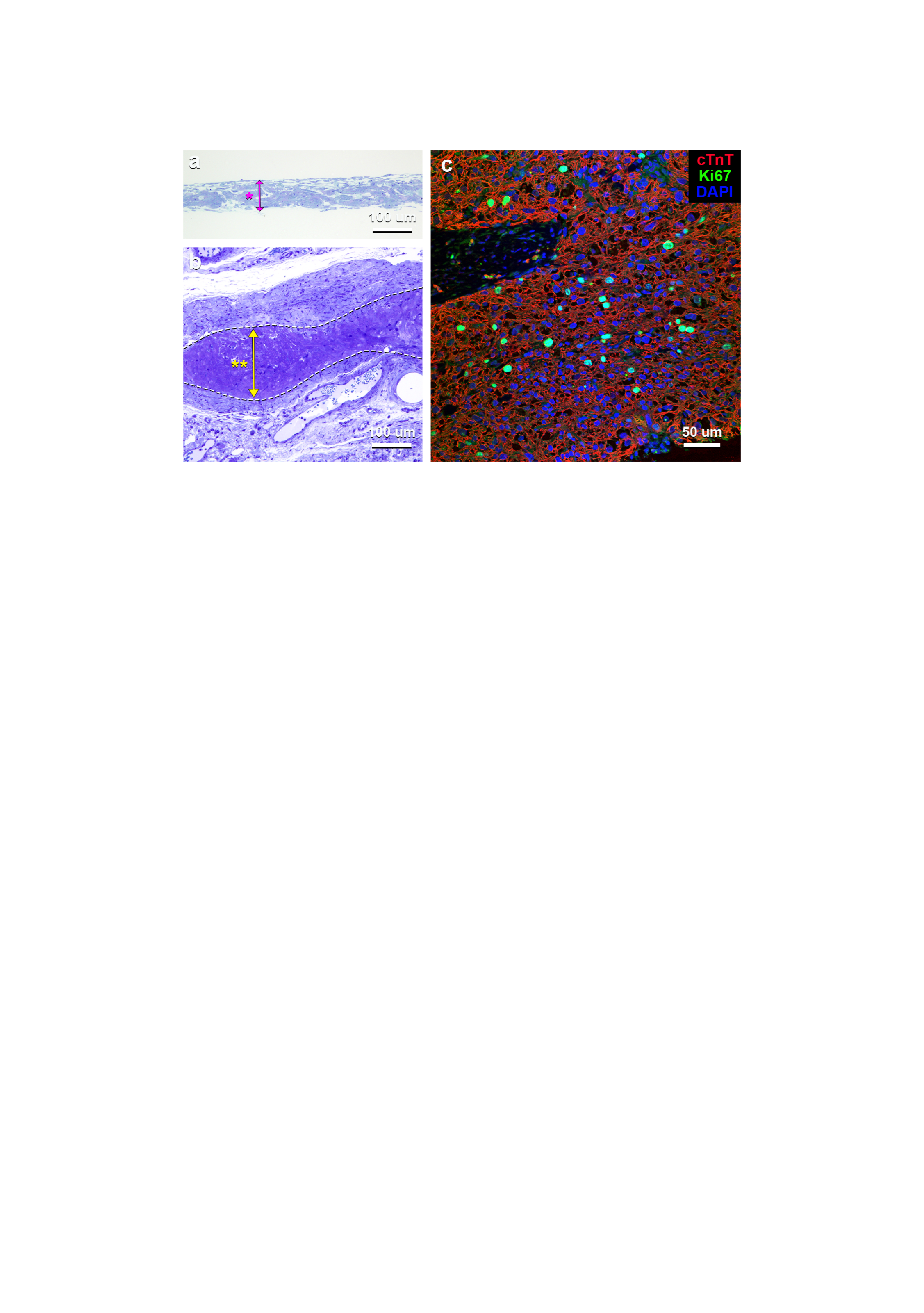
**

**Supplementary Figure 2 │Light microscopic images of tissue sections of the iPSC-CM 3D-tissues in *in vitro* (a) and 28 days after implantation to rat infarcted heart (b, c).** (**a, b**) The iPSC-CM tissue after implantation is significantly thicker (261 ± 64µm; n = 3) than before implantation (69 ± 20µm; n = 3). The broken line in **b** indicates a boundary of the implant. (**c**) Immunostaining for cTnT (red) and Ki67 (green) shows many iPSC-CMs in the implant. Cellular nuclei are stained with DAPI.


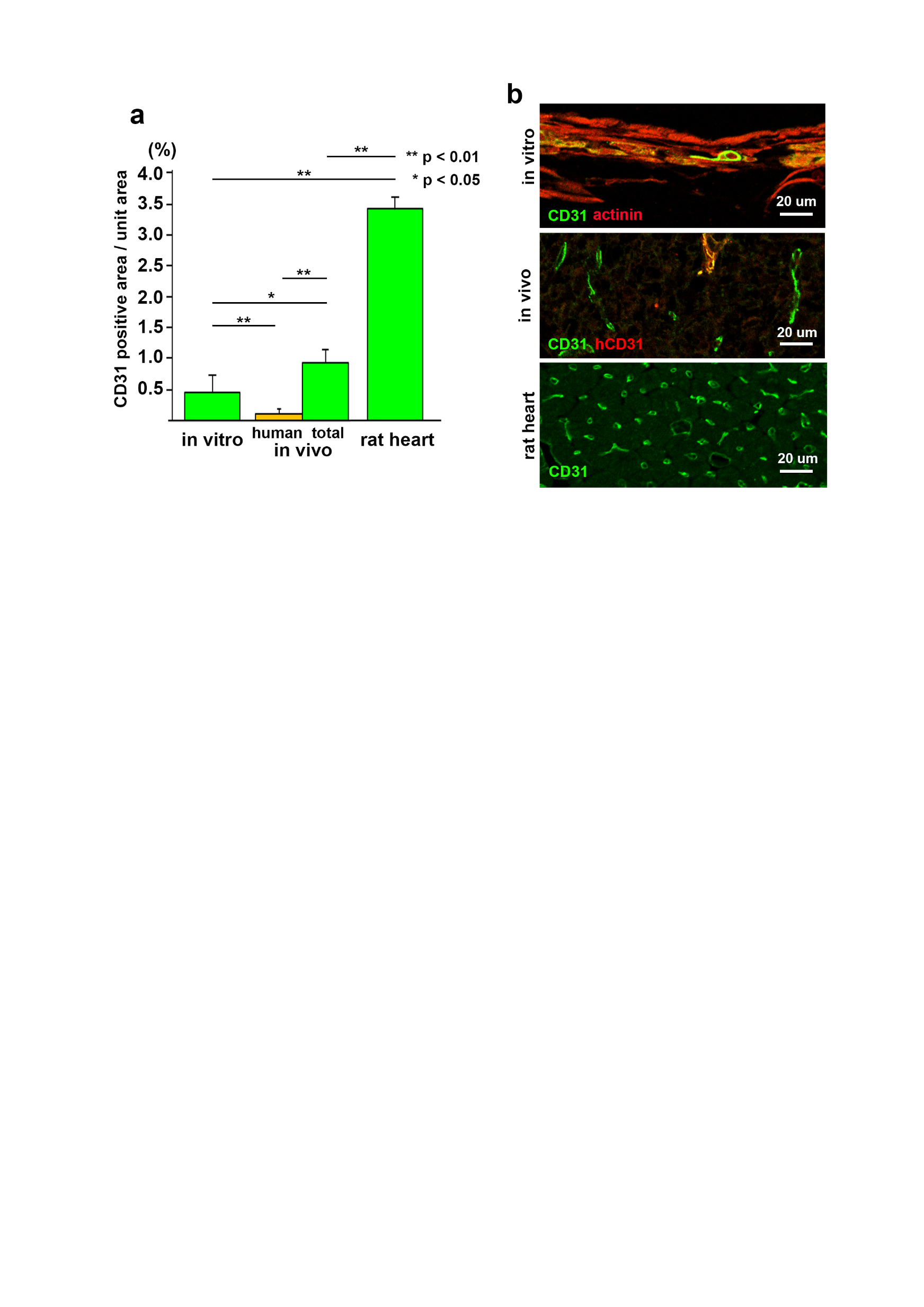


**Supplementary Figure 3 │ Comparison of vascular distribution in iPSC-CM 3D-tissues before (*in vitro*) and after implantation (*in vivo*) to rat infarcted heart and normal rat cardiac ventricle (*rat heart*).** The CD31-immunopositive vascular area of the iPSC-CM tissues before (n = 3, total: seven fields) and after implantation (n=3, total: six fields) and in the normal adult rat heart (n = 1, total: five fields) were measured using National Institutes of Health’s ImageJ software, and expressed as a percentage. These data were presented as means ± standard deviations. Comparisons of ratio of the CD31-positive-cell-area per unit area among in the tissue in each context were performed by Tukey’s test at a significance level of 0.05 using SPSS 12.0J for Windows software package (SPSS Japan Inc., Tokyo, Japan). (**a**) The green and yellow columns show the ratios of multi-species CD31-immunopositive vascular area and human CD31-immunopositive vascular area, respectively. The ratio of the multi-species CD31-immunopositive vascular area per unit area is significantly higher 28 days after implantation (*in vivo*; 0.94±0.22 %; the vessels of both rat and human origins) than before (*in vitro*; 0.49 ± 0.27 %; all the vessels of human origin), but the human CD31-immunopositivve vascular area (the vessels of human origin) shows a decrease after implantation *(in vitro*; 0.49 ± 0.27 % vs *in vivo*, human; 0.10 ± 0.08 %). The vascular area in the normal rat cardiac ventricle (rat heart; 3.4 ± 0.20 %) is significantly higher than those in the 3D-tissue before and after implantation. (**b**) Representative images of immunostaining for multi-species CD31 (green) in tissue sections of the iPSC-CM 3D-tissues before and after implantation and of normal rat cardiac ventricle. Each top and middle panel also shows iPSC-CMs immunoreactive for sarcomeric α-actinin (red) and vessels of human origin immunoreactive for human CD31 (red).

**
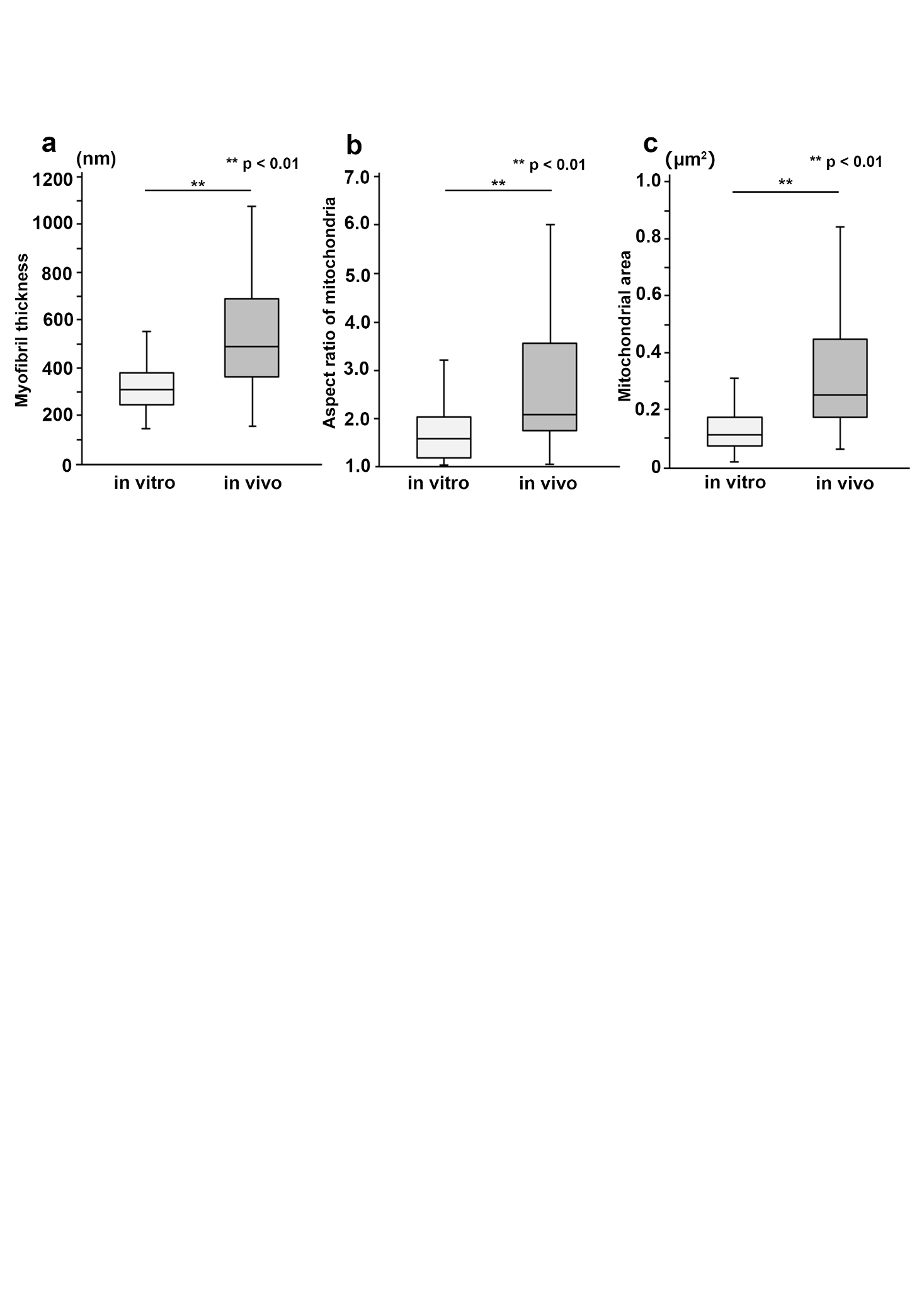
**

**Supplementary Figure 4 │ Morphometric comparison of myofibril (a) and mitochondria (b, c) within iPSC-CMs between before (*in vitro*) and after implantation (*in vivo*) to rat infarcted heart.** The myofibril thickness, mitochondrial aspect ratio and area within the iPSC-CMs before (n = 50 myofibrils and mitochondria) and after implantation (n = 50 myofibrils and mitochondria) were measured from TEM images using ImageJ, and expressed as median and interquartile range (IQR). These data were compared by SPSS 12.0J, using Mann-Whitney U test at a significance level of 0.05. (**a**) The myofibrils thickness within iPSC-CMs in *in vivo* (median 491.2 nm; IQR 365.7–666.9 nm) is significantly higher than that in *in vitro* (median 311.3 nm; IQR 243.9–380.0 nm). (**b, c**) The mitochondrial aspect ratio and area show a significant increase after implantation *(in vitro*; median 1.49; IQR 1.20–2.03 and median 0.12 µm^2^; IQR 0.08–0.17µm^2^ vs *in vivo*; median 2.10; IQR 1.76–3.41 and median 0.25 µm^2^; IQR 0.18–0.43 µm^2^, respectively).


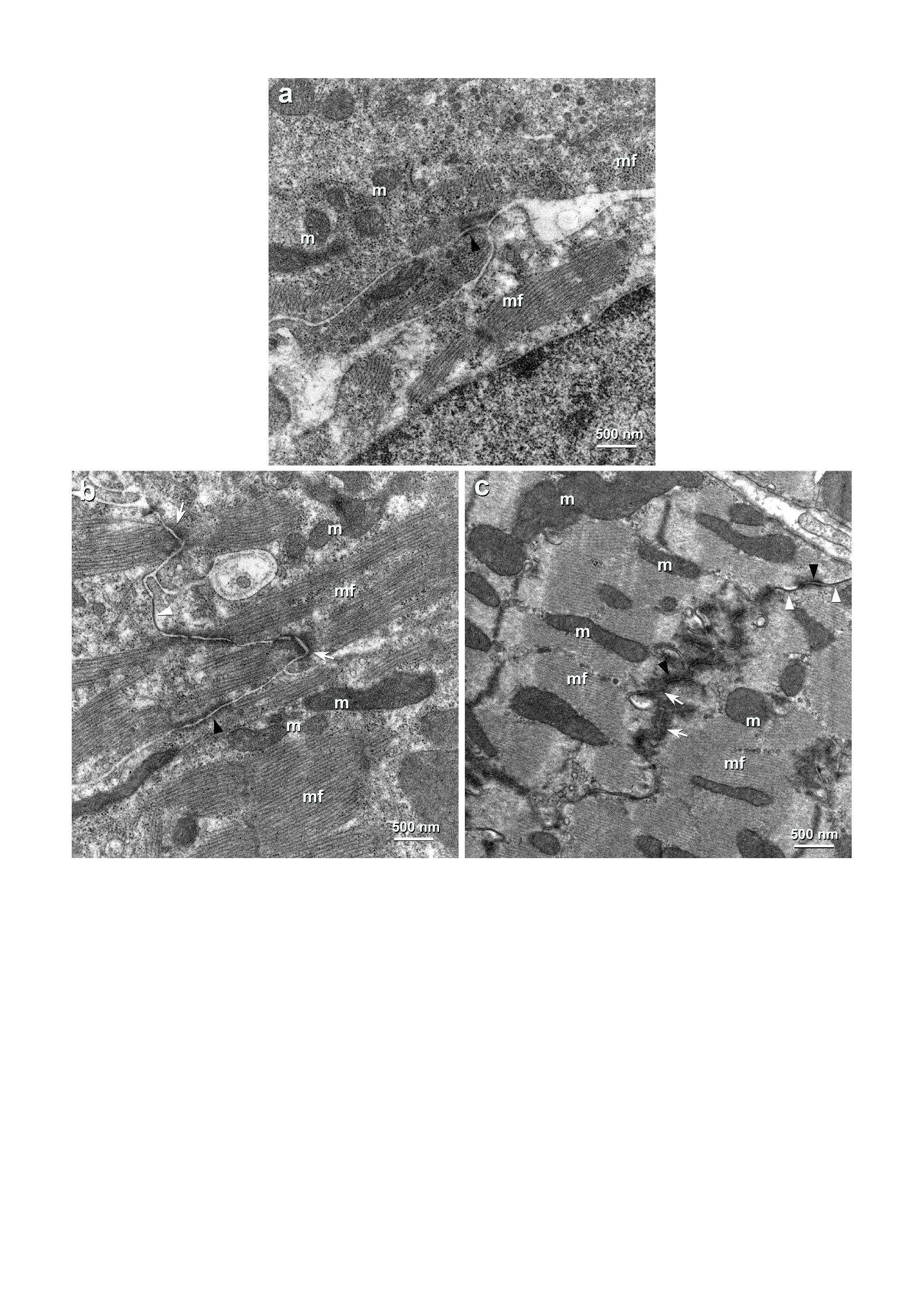


**Supplementary Figure 5 │TEM images of mammalian (rat) cardiomyocytes in embryo (a; 18.5 days post coitus), neonate (b; 0.5 days after birth) and adult (c).** (**a**) The sarcoplasm shows a sparse distribution of thin myofibrils (mf) and small mitochondria (m) with a few cristae. A formation of desmosome (arrowhead) is seen at the contact site between the cells, but the other adhesive apparatuses are obscure. (**b**) The sarcoplasm shows many ribbon-like myofibrils (mf) ranging in parallel and some oval or cylindrical mitochondria (m) with several lamellar cristae. A formation of some adherens junctions (white arrows) and desmosomes (black arrowheads), and gap junction (white arrowhead) is seen at the interdigitated contact site between the cells. (**c**) The sarcoplasm shows a dense distribution of thick bundle-like myofibrils (mf) being in parallel and large oval or cylindrical mitochondria (m) ranging along the myofibrils. The contact site between the cells shows intercalated disk containing many adherens junctions (white arrows), desmosomes (black arrowheads) and gap junctions (white arrowheads).

**
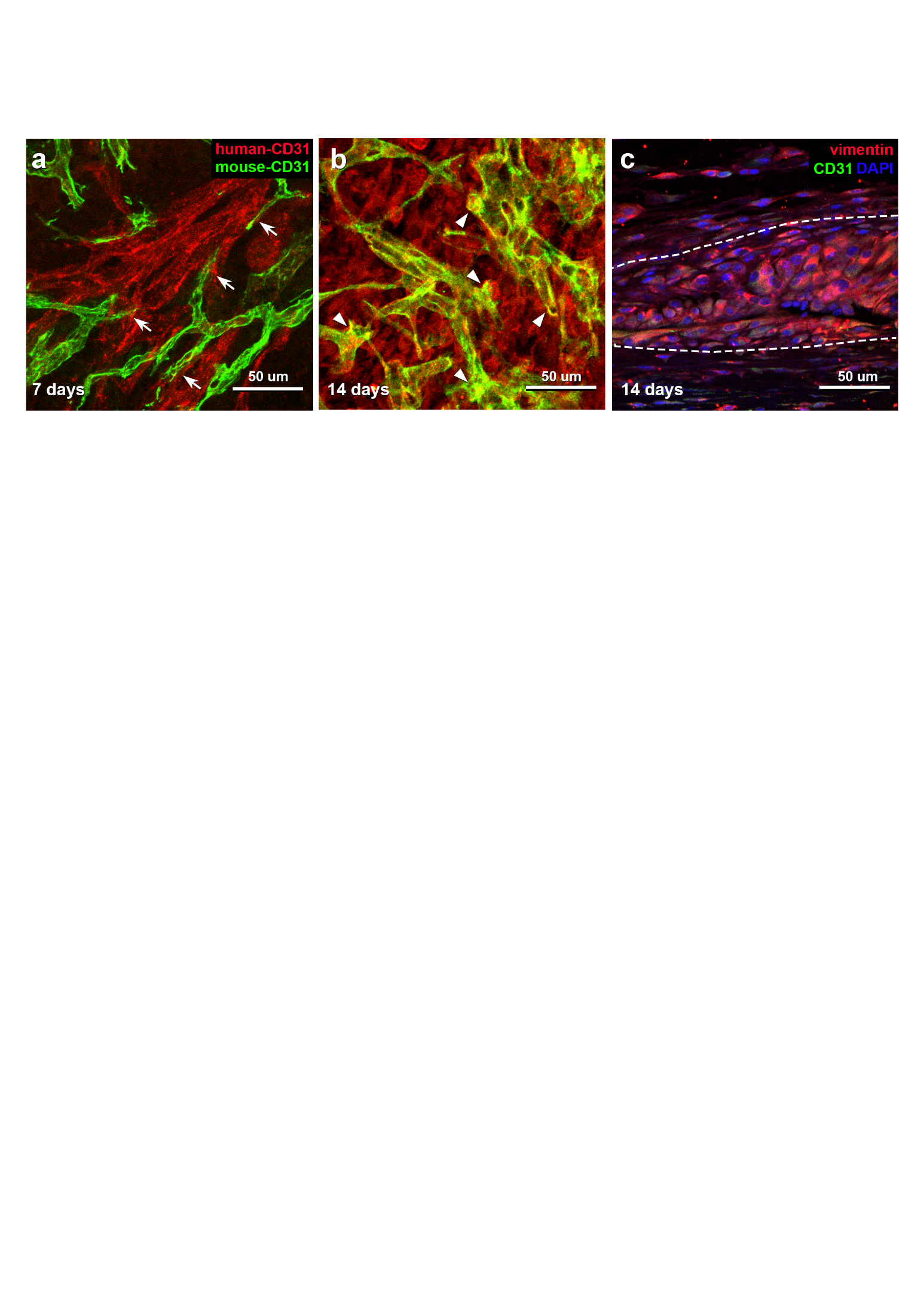
**

**Supplementary Figure 6 │Light microscopic images of tissue sections of the human 3D-tissues with (a, b) or without (c) vascular network implanted to immunodeficient mouse subcutaneous tissue.** (**a, b**) Immunostaining for human CD31 (red) and mouse CD31 (green). Close contacts (arrow in **a**) and endothelial fusions (arrowheads in **b**) between murine blood vessels extending into the implant and human vessels are seen 7 days and 14 days after implantation, respectively. (**c**) Immunostaining for vimentin (red) and multi-pieces CD31 (green). Few vascular components are seen in the implant comprising vimentin-immunopositive stromal cells 14 days after implantation. The broken line in **b** indicates a boundary of the implant.
